# 4-(3-Methyl­phen­yl)-3-phenyl-5-(2-pyrid­yl)-4*H*-1,2,4-triazole

**DOI:** 10.1107/S1600536809049174

**Published:** 2009-11-25

**Authors:** Xiaoning Gong, Zuoxiang Wang, Yan Liu

**Affiliations:** a School of Chemistry and Engineering, Southeast University, Nanjing 211189, People’s Republic of China

## Abstract

In the title compound, C_20_H_16_N_4_, the *m*-tolyl and phenyl substituents form dihedral angles of 74.20 (6) and 36.94 (8)°, respectively, with the 1,2,4-triazole ring and the dihedral angle between the triazole and pyridine rings is 36.06 (9)°. In the crystal, mol­ecules are linked by C—H⋯N and C—H⋯π inter­actions.

## Related literature

For the synthesis of the title compound, see: Klingsberg (1958 ▶). For related structures, see: Wang *et al.* (2005 ▶); Huang *et al.* (2008 ▶).
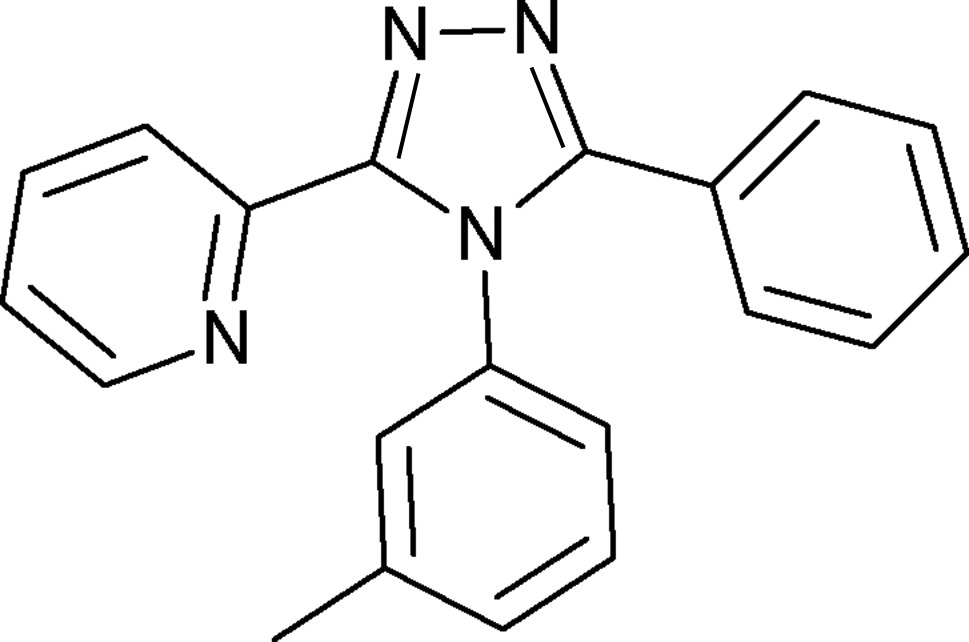


## Experimental

### 

#### Crystal data

C_20_H_16_N_4_
*M* *_r_* = 312.37
Monoclinic, 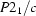

*a* = 11.246 (3) Å
*b* = 9.377 (2) Å
*c* = 18.956 (5) Å
β = 124.655 (16)°
*V* = 1644.3 (7) Å^3^
*Z* = 4
Mo *K*α radiation
μ = 0.08 mm^−1^
*T* = 293 K
0.65 × 0.50 × 0.27 mm

#### Data collection

Rigaku SCXmini diffractometer
Absorption correction: multi-scan (*CrystalClear*; Rigaku, 2005 ▶) *T*_min_ = 0.787, *T*_max_ = 1.000
16277 measured reflections
3751 independent reflections
2691 reflections with *I* > 2σ(*I*)
*R*_int_ = 0.040

#### Refinement

*R*[*F* ^2^ > 2σ(*F* ^2^)] = 0.057
*wR*(*F* ^2^) = 0.162
*S* = 1.06
3751 reflections
218 parameters
H-atom parameters constrained
Δρ_max_ = 0.31 e Å^−3^
Δρ_min_ = −0.23 e Å^−3^

### 

Data collection: *CrystalClear* (Rigaku, 2005 ▶); cell refinement: *CrystalClear*; data reduction: *CrystalClear*; program(s) used to solve structure: *SHELXS97* (Sheldrick, 2008 ▶); program(s) used to refine structure: *SHELXL97* (Sheldrick, 2008 ▶); molecular graphics: *SHELXTL* (Sheldrick, 2008 ▶); software used to prepare material for publication: *PRPKAPPA* (Ferguson, 1999 ▶).

## Supplementary Material

Crystal structure: contains datablocks I, global. DOI: 10.1107/S1600536809049174/gk2238sup1.cif

Structure factors: contains datablocks I. DOI: 10.1107/S1600536809049174/gk2238Isup2.hkl

Additional supplementary materials:  crystallographic information; 3D view; checkCIF report

## Figures and Tables

**Table 1:** Hydrogen-bond geometry (Å, °)

*D*—H⋯*A*	*D*—H	H⋯*A*	*D*⋯*A*	*D*—H⋯*A*
C12—H12⋯N2^i^	0.93	2.60	3.375 (3)	142
C20—H20*A*⋯N2^ii^	0.96	2.62	3.549 (4)	163
C10—H10⋯*Cg*1^ii^	0.93	2.72	3.646 (3)	175

Symmetry codes: (i) 
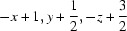
; (ii) 
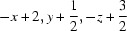
. *Cg*1 is the centroid of the C3–C8 ring.

**Fig. 1.**
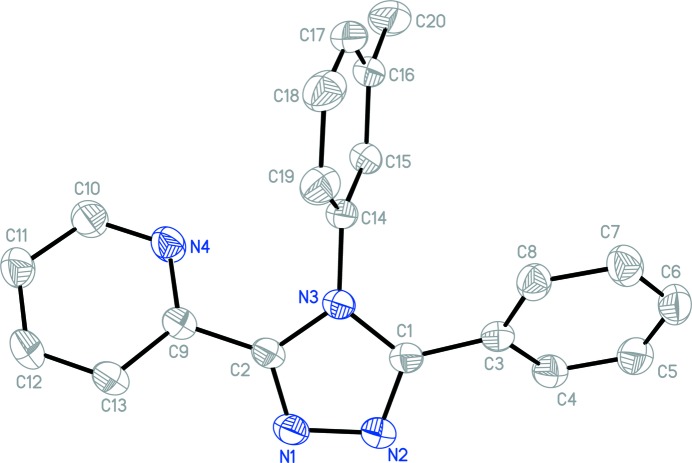
The molecular structure of the title compound with the atom labelling. Displacement ellipsoids are shown at the 30% probability level.

## supplementary crystallographic information

### Comment

Recently we have prepared some new 1,2,4-triazoles and their complexes (Wang *et al.*, 2005; Huang *et al.*, 2008). We report here the crystal structure of the title compound.

### Experimental

The title compound was prepared by the reaction of 3,3^'^– dimethylphenylphosphazoanilide (2.90, 12 mmol) with *N*-benzoyl-N^'^-(2-pyridoyl)hydrazine (2.41 g, 10 mmol) in *N*,*N*-dimethylaniline at 463–473 K for 5 hrs (Klingsberg, 1958). Single crystals suitable for X-ray diffraction were obtained by recrystallization from water.

### Refinement

Positional parameters of all the H atoms were calculated geometrically. The H atoms were allowed to ride on the C atoms to which they were bonded, riding with C—H = 0.93 Å (aromatic) and 0.96 Å (methyl); *U*_iso_(H) = 1.2 or 1.5 times *U*_eq_(C).

### Crystal data

**Table:**

C_20_H_16_N_4_	*F*(000) = 656
*M**_r_* = 312.37	*D*_x_ = 1.262 Mg m^−^^3^
Monoclinic, *P*2_1_/*c*	Mo *K*α radiation, λ = 0.71073 Å
Hall symbol: -P 2ybc	Cell parameters from 3426 reflections
*a* = 11.246 (3) Å	θ = 2.3–27.5°
*b* = 9.377 (2) Å	µ = 0.08 mm^−^^1^
*c* = 18.956 (5) Å	*T* = 293 K
β = 124.655 (16)°	Block, white
*V* = 1644.3 (7) Å^3^	0.65 × 0.50 × 0.27 mm
*Z* = 4	

### Data collection

**Table:**

Rigaku SCXmini diffractometer	3751 independent reflections
Radiation source: fine-focus sealed tube	2691 reflections with *I* > 2σ(*I*)
graphite	*R*_int_ = 0.040
ω scan	θ_max_ = 27.5°, θ_min_ = 2.3°
Absorption correction: multi-scan (*CrystalClear*; Rigaku, 2005)	*h* = −14→14
*T*_min_ = 0.787, *T*_max_ = 1.000	*k* = −12→12
16277 measured reflections	*l* = −24→24

### Refinement

**Table:**

Refinement on *F*^2^	Primary atom site location: structure-invariant direct methods
Least-squares matrix: full	Secondary atom site location: difference Fourier map
*R*[*F*^2^ > 2σ(*F*^2^)] = 0.057	Hydrogen site location: inferred from neighbouring sites
*wR*(*F*^2^) = 0.162	H-atom parameters constrained
*S* = 1.06	*w* = 1/[σ^2^(*F*_o_^2^) + (0.0823*P*)^2^ + 0.1727*P*] where *P* = (*F*_o_^2^ + 2*F*_c_^2^)/3
3751 reflections	(Δ/σ)_max_ < 0.001
218 parameters	Δρ_max_ = 0.31 e Å^−^^3^
0 restraints	Δρ_min_ = −0.23 e Å^−^^3^

### Special details

**Table:**

Geometry. All e.s.d.'s (except the e.s.d. in the dihedral angle between two l.s. planes) are estimated using the full covariance matrix. The cell e.s.d.'s are taken into account individually in the estimation of e.s.d.'s in distances, angles and torsion angles; correlations between e.s.d.'s in cell parameters are only used when they are defined by crystal symmetry. An approximate (isotropic) treatment of cell e.s.d.'s is used for estimating e.s.d.'s involving l.s. planes.
Refinement. Refinement of *F*^2^ against ALL reflections. The weighted *R*-factor *wR* and goodness of fit *S* are based on *F*^2^, conventional *R*-factors *R* are based on *F*, with *F* set to zero for negative *F*^2^. The threshold expression of *F*^2^ > σ(*F*^2^) is used only for calculating *R*-factors(gt) *etc*. and is not relevant to the choice of reflections for refinement. *R*-factors based on *F*^2^ are statistically about twice as large as those based on *F*, and *R*- factors based on ALL data will be even larger.

### Fractional atomic coordinates and isotropic or equivalent isotropic displacement parameters (Å^2^)

**Table:**

	*x*	*y*	*z*	*U*_iso_*/*U*_eq_	
C1	0.91724 (18)	−0.14398 (19)	0.88581 (11)	0.0461 (4)	
C2	0.74890 (17)	−0.02378 (18)	0.77576 (11)	0.0447 (4)	
C3	1.05742 (18)	−0.1952 (2)	0.95950 (11)	0.0481 (4)	
C4	1.0759 (2)	−0.3404 (2)	0.97830 (14)	0.0603 (5)	
H4	1.0017	−0.4038	0.9427	0.072*	
C5	1.2038 (2)	−0.3906 (2)	1.04945 (15)	0.0703 (6)	
H5	1.2151	−0.4876	1.0619	0.084*	
C6	1.3143 (2)	−0.2978 (3)	1.10189 (14)	0.0684 (6)	
H6	1.4005	−0.3317	1.1498	0.082*	
C7	1.2969 (2)	−0.1545 (2)	1.08313 (12)	0.0618 (5)	
H7	1.3719	−0.0917	1.1186	0.074*	
C8	1.16981 (19)	−0.1029 (2)	1.01265 (11)	0.0513 (4)	
H8	1.1594	−0.0058	1.0007	0.062*	
C9	0.67146 (17)	0.07496 (19)	0.70258 (10)	0.0457 (4)	
C10	0.6617 (2)	0.1881 (3)	0.59246 (13)	0.0687 (6)	
H10	0.7040	0.2101	0.5636	0.082*	
C11	0.5267 (2)	0.2440 (2)	0.56156 (13)	0.0679 (6)	
H11	0.4792	0.3009	0.5127	0.081*	
C12	0.4644 (2)	0.2140 (2)	0.60434 (13)	0.0625 (5)	
H12	0.3740	0.2507	0.5852	0.075*	
C13	0.53747 (18)	0.1287 (2)	0.67597 (12)	0.0529 (5)	
H13	0.4973	0.1074	0.7062	0.063*	
C14	1.00456 (17)	0.05403 (19)	0.83598 (10)	0.0445 (4)	
C15	1.08632 (18)	−0.0030 (2)	0.80966 (11)	0.0510 (4)	
H15	1.0713	−0.0967	0.7903	0.061*	
C16	1.19103 (19)	0.0793 (2)	0.81200 (13)	0.0595 (5)	
C17	1.2116 (2)	0.2171 (3)	0.84272 (14)	0.0676 (6)	
H17	1.2815	0.2735	0.8447	0.081*	
C18	1.1320 (2)	0.2735 (2)	0.87042 (13)	0.0693 (6)	
H18	1.1494	0.3660	0.8917	0.083*	
C19	1.0255 (2)	0.1913 (2)	0.86644 (12)	0.0583 (5)	
H19	0.9695	0.2283	0.8840	0.070*	
C20	1.2756 (3)	0.0194 (3)	0.7795 (2)	0.1004 (10)	
H20A	1.2660	0.0814	0.7363	0.151*	
H20B	1.2390	−0.0734	0.7555	0.151*	
H20C	1.3757	0.0120	0.8262	0.151*	
N1	0.68517 (15)	−0.12089 (17)	0.79297 (10)	0.0527 (4)	
N2	0.79217 (16)	−0.19790 (17)	0.86336 (10)	0.0536 (4)	
N3	0.89575 (14)	−0.03337 (15)	0.83260 (9)	0.0441 (3)	
N4	0.73453 (16)	0.1044 (2)	0.66152 (10)	0.0615 (5)	

### Atomic displacement parameters (Å^2^)

**Table:**

	*U*^11^	*U*^22^	*U*^33^	*U*^12^	*U*^13^	*U*^23^
C1	0.0450 (9)	0.0508 (9)	0.0478 (9)	−0.0015 (8)	0.0295 (8)	0.0013 (8)
C2	0.0375 (9)	0.0539 (9)	0.0450 (9)	−0.0015 (7)	0.0247 (8)	−0.0039 (8)
C3	0.0455 (9)	0.0578 (10)	0.0489 (9)	0.0031 (8)	0.0316 (8)	0.0057 (8)
C4	0.0523 (11)	0.0593 (12)	0.0703 (12)	−0.0002 (9)	0.0354 (10)	0.0096 (10)
C5	0.0653 (13)	0.0683 (13)	0.0807 (15)	0.0140 (11)	0.0436 (12)	0.0243 (12)
C6	0.0563 (12)	0.0908 (16)	0.0562 (11)	0.0201 (11)	0.0308 (10)	0.0193 (11)
C7	0.0533 (11)	0.0786 (14)	0.0473 (10)	0.0022 (10)	0.0250 (9)	−0.0035 (10)
C8	0.0529 (10)	0.0559 (10)	0.0445 (9)	0.0026 (8)	0.0273 (9)	0.0014 (8)
C9	0.0408 (9)	0.0534 (10)	0.0433 (9)	−0.0030 (7)	0.0242 (8)	−0.0071 (7)
C10	0.0587 (12)	0.0980 (16)	0.0542 (11)	0.0093 (12)	0.0349 (10)	0.0124 (11)
C11	0.0525 (12)	0.0875 (15)	0.0519 (11)	0.0106 (10)	0.0226 (10)	0.0141 (11)
C12	0.0395 (10)	0.0748 (13)	0.0626 (12)	0.0098 (9)	0.0227 (9)	0.0021 (10)
C13	0.0409 (9)	0.0652 (11)	0.0542 (10)	−0.0010 (8)	0.0280 (8)	−0.0045 (9)
C14	0.0375 (9)	0.0542 (10)	0.0400 (8)	−0.0046 (7)	0.0210 (7)	0.0040 (7)
C15	0.0403 (9)	0.0629 (11)	0.0487 (10)	−0.0006 (8)	0.0245 (8)	0.0053 (8)
C16	0.0403 (10)	0.0791 (14)	0.0584 (11)	0.0024 (9)	0.0277 (9)	0.0208 (10)
C17	0.0481 (11)	0.0823 (15)	0.0605 (12)	−0.0146 (10)	0.0237 (10)	0.0190 (11)
C18	0.0746 (14)	0.0596 (12)	0.0589 (12)	−0.0203 (10)	0.0292 (11)	−0.0003 (10)
C19	0.0645 (12)	0.0581 (11)	0.0536 (11)	−0.0049 (9)	0.0344 (10)	0.0005 (9)
C20	0.0799 (17)	0.123 (2)	0.132 (2)	0.0221 (16)	0.0802 (18)	0.047 (2)
N1	0.0420 (8)	0.0610 (9)	0.0584 (9)	−0.0034 (7)	0.0304 (7)	−0.0005 (8)
N2	0.0450 (8)	0.0583 (9)	0.0599 (9)	−0.0028 (7)	0.0314 (8)	0.0045 (7)
N3	0.0379 (7)	0.0515 (8)	0.0450 (7)	−0.0017 (6)	0.0249 (6)	0.0015 (6)
N4	0.0491 (9)	0.0881 (12)	0.0516 (9)	0.0111 (8)	0.0313 (8)	0.0086 (8)

### Geometric parameters (Å, °)

**Table:**

C1—N2	1.317 (2)	C11—C12	1.368 (3)
C1—N3	1.369 (2)	C11—H11	0.9300
C1—C3	1.470 (2)	C12—C13	1.375 (3)
C2—N1	1.310 (2)	C12—H12	0.9300
C2—N3	1.368 (2)	C13—H13	0.9300
C2—C9	1.472 (2)	C14—C19	1.375 (3)
C3—C8	1.383 (3)	C14—C15	1.379 (2)
C3—C4	1.393 (3)	C14—N3	1.444 (2)
C4—C5	1.379 (3)	C15—C16	1.388 (2)
C4—H4	0.9300	C15—H15	0.9300
C5—C6	1.373 (3)	C16—C17	1.381 (3)
C5—H5	0.9300	C16—C20	1.504 (3)
C6—C7	1.375 (3)	C17—C18	1.375 (3)
C6—H6	0.9300	C17—H17	0.9300
C7—C8	1.375 (3)	C18—C19	1.390 (3)
C7—H7	0.9300	C18—H18	0.9300
C8—H8	0.9300	C19—H19	0.9300
C9—N4	1.346 (2)	C20—H20A	0.9600
C9—C13	1.384 (2)	C20—H20B	0.9600
C10—N4	1.335 (3)	C20—H20C	0.9600
C10—C11	1.383 (3)	N1—N2	1.387 (2)
C10—H10	0.9300		
			
N2—C1—N3	110.12 (15)	C13—C12—H12	120.5
N2—C1—C3	123.56 (16)	C12—C13—C9	119.16 (18)
N3—C1—C3	126.29 (15)	C12—C13—H13	120.4
N1—C2—N3	110.15 (15)	C9—C13—H13	120.4
N1—C2—C9	123.91 (15)	C19—C14—C15	121.67 (16)
N3—C2—C9	125.86 (15)	C19—C14—N3	119.22 (16)
C8—C3—C4	118.92 (17)	C15—C14—N3	119.10 (16)
C8—C3—C1	122.00 (17)	C14—C15—C16	120.07 (19)
C4—C3—C1	119.04 (17)	C14—C15—H15	120.0
C5—C4—C3	120.3 (2)	C16—C15—H15	120.0
C5—C4—H4	119.9	C17—C16—C15	117.99 (19)
C3—C4—H4	119.9	C17—C16—C20	121.9 (2)
C6—C5—C4	120.2 (2)	C15—C16—C20	120.1 (2)
C6—C5—H5	119.9	C18—C17—C16	122.09 (18)
C4—C5—H5	119.9	C18—C17—H17	119.0
C5—C6—C7	119.64 (19)	C16—C17—H17	119.0
C5—C6—H6	120.2	C17—C18—C19	119.6 (2)
C7—C6—H6	120.2	C17—C18—H18	120.2
C8—C7—C6	120.8 (2)	C19—C18—H18	120.2
C8—C7—H7	119.6	C14—C19—C18	118.5 (2)
C6—C7—H7	119.6	C14—C19—H19	120.7
C7—C8—C3	120.15 (19)	C18—C19—H19	120.7
C7—C8—H8	119.9	C16—C20—H20A	109.5
C3—C8—H8	119.9	C16—C20—H20B	109.5
N4—C9—C13	122.49 (17)	H20A—C20—H20B	109.5
N4—C9—C2	116.73 (15)	C16—C20—H20C	109.5
C13—C9—C2	120.72 (16)	H20A—C20—H20C	109.5
N4—C10—C11	123.52 (19)	H20B—C20—H20C	109.5
N4—C10—H10	118.2	C2—N1—N2	107.66 (14)
C11—C10—H10	118.2	C1—N2—N1	107.09 (14)
C12—C11—C10	118.65 (19)	C2—N3—C1	104.97 (13)
C12—C11—H11	120.7	C2—N3—C14	127.64 (14)
C10—C11—H11	120.7	C1—N3—C14	127.40 (13)
C11—C12—C13	119.03 (18)	C10—N4—C9	117.14 (16)
C11—C12—H12	120.5		

### Hydrogen-bond geometry (Å, °)

**Table:**

*D*—H···*A*	*D*—H	H···*A*	*D*···*A*	*D*—H···*A*
C12—H12···N2^i^	0.93	2.60	3.375 (3)	142
C20—H20A···N2^ii^	0.96	2.62	3.549 (4)	163
C10—H10···Cg1^ii^	0.93	2.72	3.646 (3)	175

Symmetry codes: (i) −*x*+1, *y*+1/2, −*z*+3/2; (ii) −*x*+2, *y*+1/2, −*z*+3/2.

## References

[bb1] Ferguson, G. (1999). *PRPKAPPA*. University of Guelph, Canada.

[bb2] Huang, L., Wang, Z., Zhang, X. & Wu, P. (2008). *Acta Cryst.* E**64**, m741–m742. 10.1107/S1600536808012026 PMC2961222 21202261

[bb3] Klingsberg, E. (1958). *J. Org. Chem* **23**, 1086–1087.

[bb4] Rigaku (2005). *CrystalClear*. Rigaku Corporation, Tokyo, Japan.

[bb5] Sheldrick, G. M. (2008). *Acta Cryst.* A**64**, 112–122. 10.1107/S0108767307043930 18156677

[bb6] Wang, Z.-X., Lan, Y., Yuan, L.-T. & Liu, C.-Y. (2005). *Acta Cryst.* E**61**, o2033–o2034.

